# Supplementary material for: T3 Critically Affects the Mhrt/Brg1 Axis to Regulate the Cardiac MHC Switch: Role of an Epigenetic Cross-Talk
Source: Cells. 2020 Sep 24;9(10):2155. doi: 10.3390/cells9102155 (PMC7598656; doi:10.3390/cells9102155)
Supplement: Supplementary file 1 [file cells-09-02155-s001.pdf]

Supplementary material

Supplementary table1. TH level in rat serum at baseline and at the end of each experimental procedure.

Model	Group	FT3 (pg/ml)		FT4 (pg/ml)	
Hyperthyroidism		*Basal*	*6 d*	*Basal*	*6 d*
	EuT	2.9±0.2	3.1±0.2	11.0±1.0	12.3±0.4
	HyperT	3.2±0.2	**13.5±2.2**	11.7±0.5	**47.3±2.1**
IR 3d endpoint		*Basal*	*3 d*	*Basal*	*3 d*
	Sham	3.1±0.3	2.8±0.2	10.9±0.7	11.3±0.8
	IR	2.9±0.3	**1.8±0.2**	11.3±1.0	9.8±0.3
	IRT3	2.8±0.3	2.9±0.3	11.8±1.2	11.9±0.4
IR 14d endpoint		*Basal*	*14 d*	*Basal*	*14 d*
	Sham	3.1±0.6	3.2±0.1	11.0±0.7	12.7±1.1
	IR	3.2±0.3	3.2±0.1	10.9±1.0	10.4±0.4
	IRT3	2.8±0.2	3.0±0.2	11.7±0.9	11.5±1.2

Values are mean±SEM, n≥4 in each group. EuT= Euthyroid group; HyperT= Hyperthyroid group; Sham= sham operated control group; IR= untreated ischemia/reperfusion group; IRT3= ischemia reperfusion group treated with T3 replacement at 3µg/kg/die. In bold: $p<0.001$ vs respective control group and basal value.

Figure S1. Pairwise alignment of the mmu Mhrt coding sequence (upper lines NR_033497.1) with the homologous, newly identified, rno Mhrt coding sequence (lower lines, BK013310). Red and green sequences refer to the forward and reverse primers used in the qRT-PCR analysis; the blue sequence identifies the binding position of the siRNA used for the silencing of Mhrt.

```
Query  1    AGCCCTACAGTCTGATGAACATTCTAGAGTATGTGGA-CACAGATGGACGCTCTGGCCAC  59
            |||||| ||||||||||||| |||||||||| ||||| | ||||||||||||||||||||
Sbjct  1    AGCCCTGCAGTCTGATGAACGTTCTAGAGTAAGTGGACCCCAGATGGACGCTCTGGCCAC  60

Query  60   AGCTTGTTGACCTGGGACTCGGCGATGTCCGCCCTCTCCTCCGCCTCATCCAGCTCGTGC  119
            ||||||||||||||||||||||| ||||||||||||||||| ||||||||||||||||||
Sbjct  61   AGCTTGTTGACCTGGGACTCGGCAATGTCCGCCCTCTCCTCTGCCTCATCCAGCTCGTGC  120

Query  120  TGCACCTTGCGGAACTTGGACAGCCTCCAGCTCATTCTCCAGCTCCCGGACCCGGGCCTC  179
            ||||||||||||||||||||||||||||||||||||||||||||||||||||||||||||
Sbjct  121  TGCACCTTGCGGAACTTGGACAGCCTCCAGCTCATTCTCCAGCTCCCGGACCCGGGCCTC  180

Query  180  CAGCTTCTGCAGCTGCTTCTTGCCGCCCTTGAGGGCGATCTGCTCTGCCTCGTCCAGACG  239
            |||||||||||||||||||||||| |||||||||||||||||||||||||||||||| ||
Sbjct  181  CAGCTTCTGCAGCTGCTTCTTGCCACCCTTGAGGGCGATCTGCTCTGCCTCGTCCAGCCG  240

Query  240  GTGCTGCAAGTCCTTGATGGTCTGCTCCATGTTCTTCTTCATGCGCTCCAGGTCGTCATT  299
            |||||||| |||||||||||||||||||||||||||||||||||||||||||||||||||
Sbjct  241  GTGCTGCAGGTCCTTGATGGTCTGCTCCATGTTCTTCTTCATGCGCTCCAGGTCGTCATT  300

Query  300  GGCACGGACAGCATCATCCAGCTGGATTTGAGTGTCCTGAGGATCAGAAAAATGAGTGGC  359
            ||||||||| || |||||||||||||||||||||||||||||| ||||||||||||||||
Sbjct  301  GGCACGGACTGCGTCATCCAGCTGGATTTGAGTGTCCTGAGGACCAGAAAAATGAGTGGC  360

Query  360  CTCATTGCGGCTGCGTGTCTCCGCATCCAGGGAGGTCTGCAGGGAGTCCACCATCCGCAG  419
            ||| ||||||||||| ||||| ||||||||||||||||||||||||||||||| ||||||
Sbjct  361  CTCGTTGCGGCTGCGCGTCTCGGCATCCAGGGAGGTCTGCAGGGAGTCCACCACCCGCAG  420

Query  420  GTGGTTGCGCTTGGCCTGCTCCATCTCCTCATCCTTCTCTGCCAGCTTCCTTTCAATCTC  479
            |||||||||||||||||||||||||||||| ||||||||||||||||||||||| |||||
Sbjct  421  GTGGTTGCGCTTGGCCTGCTCCATCTCCTCGTCCTTCTCTGCCAGCTTCCTTTCGATCTC  480

Query  480  TGCCTTGATCTGGTTGAACTCTAGCTGGGCGCGGAGGATCTTGCCCTCCTCGTGCTCCAG  539
            ||||||||||||||||||||| |||||||| |||||||||||||||||||| ||||||||
Sbjct  481  TGCCTTGATCTGGTTGAACTCCAGCTGGGCTCGGAGGATCTTGCCCTCCTCATGCTCCAG  540

Query  540  GGAGGCCTGG-AA--A-G-G--AT-ATAGATTTTGCAGGCATATAGTCAGAGACCAGGGT  591
            |||||||||| ||  | | |  |  | ||||||||||||| || |||| |||||||||
Sbjct  541  GGAGGCCTGGGAAGGAAGCGGGAGGAAAGATTTTGCAGGCTTAGAGTCGGAGACCAGGAG  600

Query  592  GGAAGCAAGGGTGTGTCTAAAAACCATGGCACAGAGAGCATTTGGGGATGGTATACATGA  651
            |||||||||||||     |||| |||||  ||||||||||||  || ||||||||||||
Sbjct  601  GGAAGCAAGGGTG-----AAAA-CCATGCTACAGAGAGCATTCAGGAATGGTATACATGG  654

Query  652  CTCAGT-AGGAGATGCAGTGGAAGGAAATGAGAAAGAGTGTGCACAAGAGAAATGAAAGC  710
            |||||| ||| | ||||| ||||||||| |||  ||||||||||||||||||||||||||
Sbjct  655  CTCAGTCAGGGG-TGCAGAGGAAGGAAAGGAG--AGAGTGTGCACAAGAGAAATGAAAGC  711

Query  711  AAGCTGAAGAGAAGGGGATGCAGACTCCCAGGGGGGCGGAGGGAGTCAGCTTTGAAGACA  770
            ||| |||||  ||||||||||||||||||||||||| |||||||| ||||||||||||||
Sbjct  712  AAGATGAAGGAAAGGGGATGCAGACTCCCAGGGGGGTGGAGGGAGCCAGCTTTGAAGACA  771

Query  771  AAGAGGAAAATGAAAAGTGTTGCCAAGGAAACAGAGGCAATGAAGCAGAGAGTAAAGG  828
            ||||||||||||||  ||||||||||||||||||||||||| ||||| | |||  |
Sbjct  772  AAGAGGAAAATGAAGTGTGTTGCCAAGGAAACAGAGGCAATCAAGCACACAGTGTAAA  829
```

Supplementary table2. List of oligonucleotide sequences.

Procedure	Type	Sequence	Accession
Transfection (NRCM)	SiRNA-Mhrt	5′- GAGUGUGCACAAGAGAAAU-3′<br>5′- AUUUCUCUUGUGCACACUC-3′	
	208a-mimic	5′-AUAAGACGAGCAAAAAGCUUGUUU-3′<br>5′-ACAAGCUUUUUGCUCGUCUUCUUU-3′	
	Cnt-mimic	5′-CUCUAGGUUAAACUCCUGGUU-3′<br>5′- AACCAGGAGUUUAACCUAAUGUU-3′	
Q-PCR	Mhrt primers	F. 5′-GCTGGGCTCGGAGGATCTTGC-3′<br>R. 5′ACTGAGCCATGTATACCATTCC-3′	BK013310
	Myh6 primers	F. 5′- GCCAAGAGCCGTGACATT-3′<br>R. 5′-TTTATTGTGGGATAGC AACAGC-3′	NM_017239.2
	Myh7 primers	F. 5′- ACGTGCTGGGCTTCACTC-3′<br>R. 5′-CCACTT TGACTCTAGGATGG-3′	NM_017240.2
	Brg1 primers	F. 5′- AGAAGGAACGGATGCGAAG-3′<br>R. 5′- GG TCAATGAGCTTGCGGTAG-3′	NM_134368.1
	Hprt primers	F. 5′- CCCAGCGTCGTGATTAGTGATG-3′<br>R. 5′- ATTTAAAAGGA ACGGTTGACA-3′	NM_012583.2
	Hmbs primers	F. 5′- TCTAGATGGCTCAGA TAGCATGCA -3′<br>R. 5′- TGGACCATCTTCTTGCTGAACA -3′	NM_013168.2
	Gapdh primers	F. 5′-GCCTTCCGTGTTCCTACCC -3′<br>R. 5′-TGCCTGCTTCACCACCT-3′	NM_017008.4
Full promoter amplification	Mhrt primers	F. 5′- AAGAGCCCTACAGTCTGATGAACA -3′<br>R. 5′- CCTTCACACAAACATTTTATTT-3′	
Site A mutagenesis	Mhrt primers	F. 5′- GGTCAGCTTGGGTCGGTA CCAAGAGACAAAGGCCAGG -3′<br>R. 5′- CCTGGCCTTTGTCTCTTGGTACCGACCC AAGCTGACC -3′	
Site B mutagenesis	Mhrt primers	F. 5′- CAGCTGTGTCCCTGGATATCGCTTGAGGCTCCT G -3′<br>R. 5′-CAGGAGCCTCAAGCGATATCCAGGGACACAGCTG-3′	

Supplementary table3. Predicted interactions between the T3-upregulated miRNAs and the 5′UTR, CDS and 3′UTR sequences of the rat and human Brg1 mRNA. For each target region the number of different miRWalk algorithms identifying a binding match is reported.

miRNA	Species	5′UTR	CDS	3′UTR	Total algorithms
338-3p	rno	1	4	1	6
	hsa	0	6	3	9
133a-3p	rno	1	1	3	5
	hsa	1	2	2	5
133b	rno	1	1	3	5
	hsa	1	2	1	4
208a-5p	rno	1	2	1	4
	hsa	2	1	2	5
208b-3p	rno	1	2	1	4
	hsa	1	2	4	7
